# Supplementary material for: Hsc70 promotes anti-tumor immunity by targeting PD-L1 for lysosomal degradation
Source: Nat Commun. 2024 May 18;15:4237. doi: 10.1038/s41467-024-48597-3 (PMC11102475; doi:10.1038/s41467-024-48597-3)
Supplement: Supplementary file 1 — Supplementary Information [file 41467_2024_48597_MOESM1_ESM.pdf]

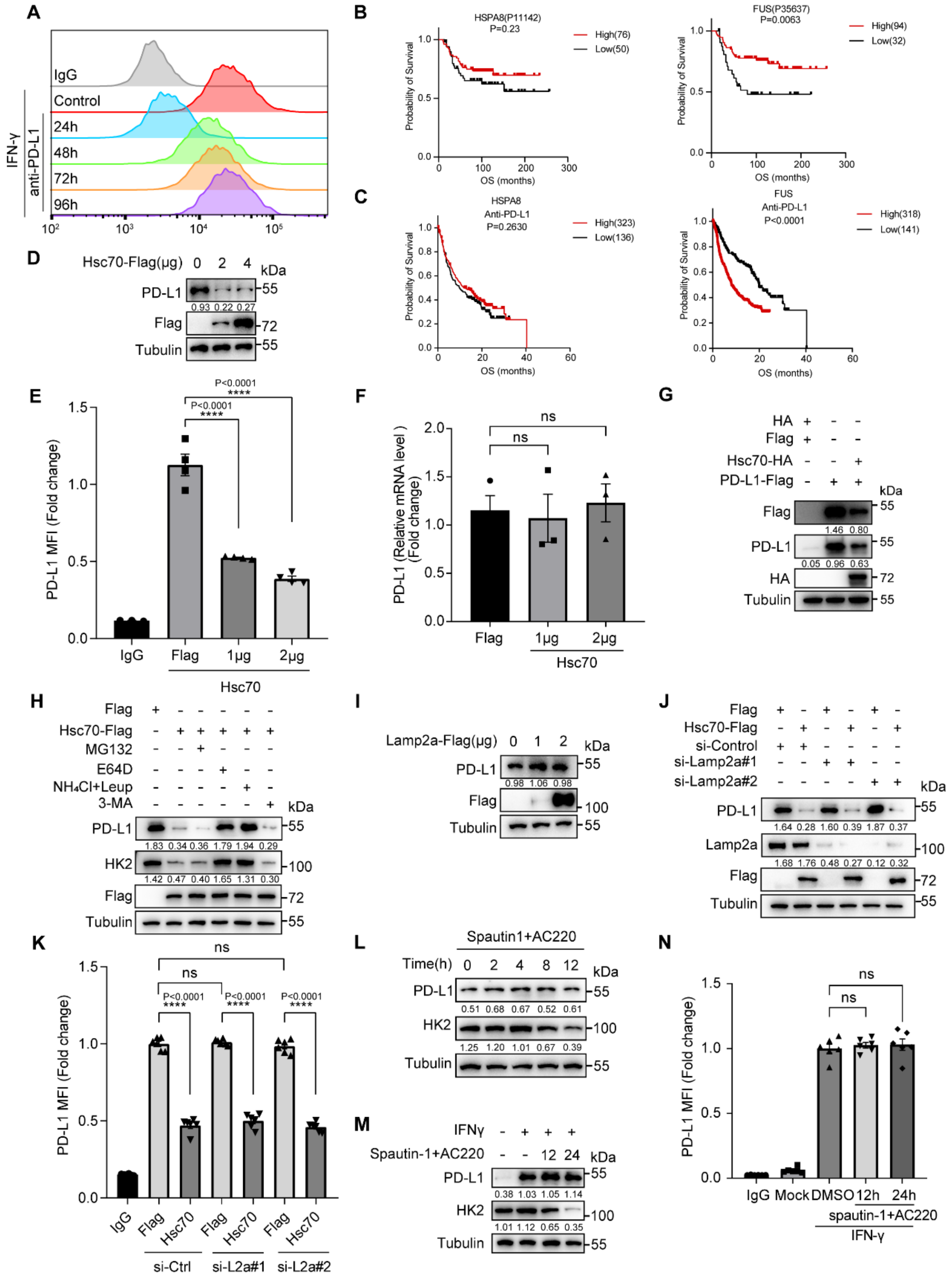

Supplementary Figure 1 Hsc70 promotes lysosomal degradation of PD-L1

(A) 4T1 cells were pretreated with IFN-γ (100 ng/mL) and treated with anti-PD-L1 Ab (100 nM) for 24 h, 48 h, 72 h and 96 h, fluorescence of PD-L1 on the surface of cell membrane was analyzed by flow cytometry.

(B) Correlation analysis between HSPA8 and FUS protein levels with overall survival

(OS) in breast cancer patients from the Kaplan-Meier Plotter platform (Kaplan-Meier plotter [proteomics] (kmplot.com))

(C) Correlation analysis between HSPA8 and FUS levels with overall survival (OS) in all tumor types of patients who have received anti-PD-L1 immune checkpoint inhibitors treatment from the Kaplan-Meier Plotter platform (Kaplan-Meier plotter [proteomics] (kmplot.com))

(D) PANC1 cells were transfected with 2 μg or 4 μg Hsc70-Flag for 24 h. PD-L1 levels were detected by western blotting.

(E) MCF-7 cells were transfected with 1 μg and 2 μg Hsc70-Flag for 24 h, fluorescence of PD-L1 on the surface of cell membrane was analyzed by flow cytometry, n=4.

(F) MCF7 cells were transfected with 1 μg or 2 μg Hsc70-Flag for 24 h, RNA was extracted and PD-L1 expression was detected using q-PCR, n=3, ns: not significance.

(G) HET293T cells were transfected with indicated plasmids for 24 h, cell lysates were immunoblotted with indiacted antibodies.

(H) PANC1 cells were transfected with indicated plasmids for 12 h, treated with or without MG132 (10 μM), NH_4Cl (20 mM), Leupeptin (100 nM), 3-MA (5 mM), E64D (10 μM) for another 12 h. Cell lysates were immunoblotted with indicated antibodies.

(I) PANC1 cells were transfected with 1 μg or 2 μg Lamp2a-Flag for 24 h. PD-L1 levels were detected by western blotting.

(J) PANC1 cells were transfected with siRNAs of Lamp2a for 36 h, transfected with or without Hsc70-Flag for another 24 h. Cell lysates were immunoblotted with indicated antibodies.

(K) MCF-7 cells were transfected with siRNAs of Lamp2a for 36 h, transfected with or without Hsc70-Flag for another 24 h, fluorescence of PD-L1 on the surface of cell membrane was analyzed by flow cytometry, n=6, ns: not significance.

(L) PANC1 cells were treated with Spautin-1 (10 μM) and AC220 (2 μM) for 0, 2, 4, 8 and 12 h. Cell lysates were immunoblotted with indicated antibodies.

(M, N) U937 cells were pretreatment with IFN-γ for 48 h, then treated with Spautin-1 (10 μM) and AC220 (2 μM) for another 12 h and 24 h. Cell lysates were immunoblotted with indicated antibodies (M), fluorescence of PD-L1 on the surface of cell membrane was analyzed by flow cytometry, n=6, ns: not significance (N).

For E, K and N, MCF-7 or U937 cells were seeded in 48-well plate with 4 or 6 replicates per group and subjected to the corresponding treatment, for F, MCF-7 cells were seeded in 6-well plate with 3 replicates per group and subjected to the corresponding treatment, these all repeated independently three times and similar results were obtained. Data shown in D, G-J, and L-M were repeated independently three times with similar results.

Data represent Mean±SEM, for E, F, K and N data, two-sided with adjustment of Tukey's multiple comparisons, one-way ANOVA, P value is indicated in the graph.

The numbers under the blots represent the value (the ratio to Tubulin) of grayscale quantification. Source data are provided as a Source Data file.

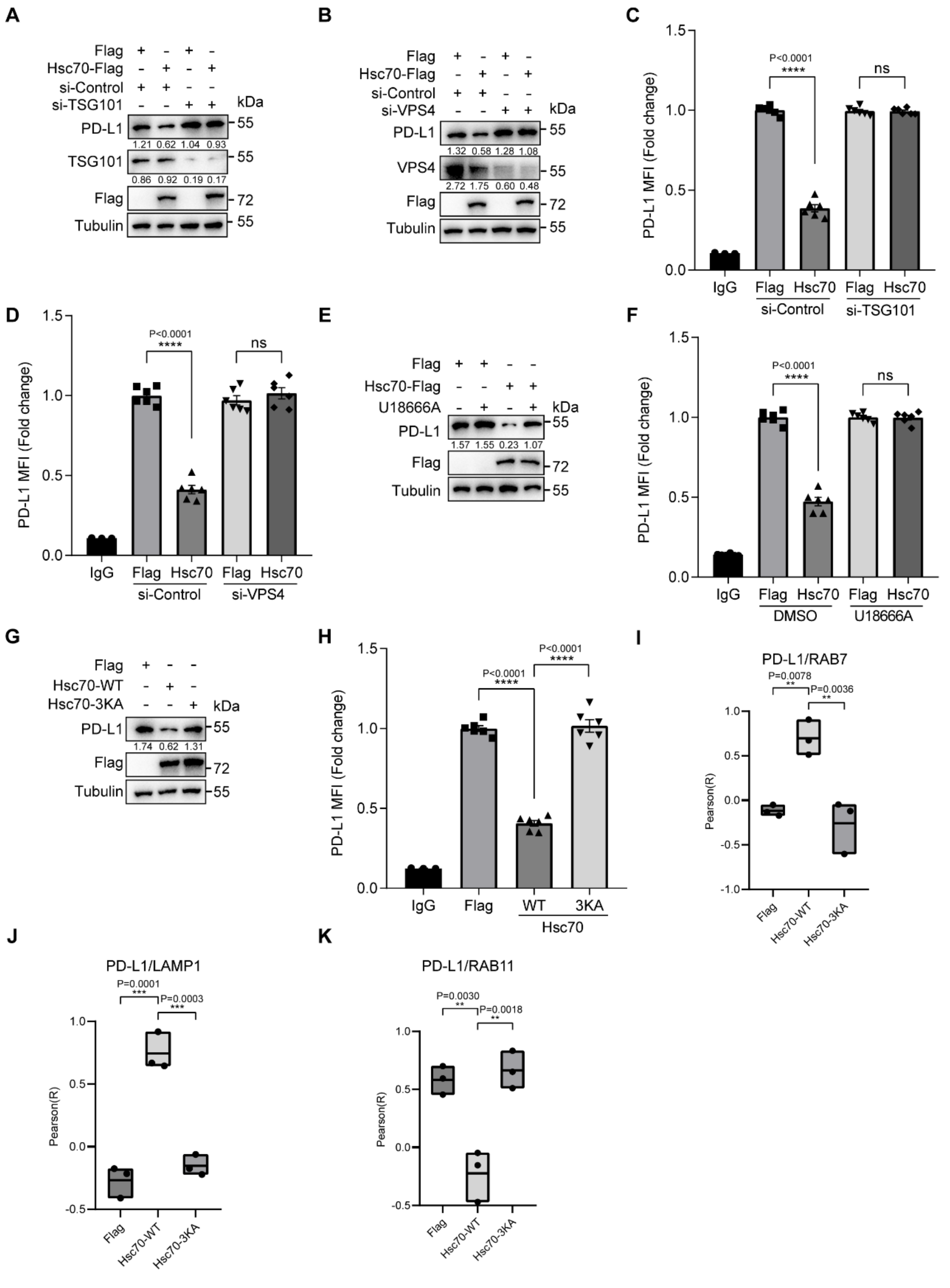

Supplementary Figure 2 Hsc70 induces PD-L1 degradation via EMI

(A) PANC1 cells were transfected with siRNAs of TSG101 for 36 h, transfected with or without Hsc70-Flag for another 24 h. Cell lysates were immunoblotted with indicated antibodies.

(B) PANC1 cells were transfected with siRNAs of VPS4 for 36 h, transfected with or

without Hsc70-Flag for another 24 h. Cell lysates were immunoblotted with indicated antibodies.
(C) MCF-7 cells were transfected with siRNAs of TSG101 for 36 h, transfected with or without Hsc70-Flag for another 24 h. Fluorescence of PD-L1 on the surface of cell membrane was analyzed by flow cytometry, n=6, ns: not significance.
(D) MCF-7 cells were transfected with siRNAs of VPS4 for 36 h, transfected with or without Hsc70-Flag for another 24 h. Fluorescence of PD-L1 on the surface of cell membrane was analyzed by flow cytometry, n=6, ns: not significance.
(E) PANC1 cells were transfected with Flag or Hsc70-Flag plasmids for 12 h, treated with or without U18666A (5 μg/mL) for another 12 h. PD-L1 levels were detected by western blotting.
(F) MCF-7 cells were transfected with Flag or Hsc70-Flag for 12 h, treated with or without U18666A (5 μg/mL) for another 12 h. Fluorescence of PD-L1 on the surface of cell membrane was analyzed by flow cytometry, n=6, ns: not significance.
(G) PANC1 cells were transfected with Hsc70-WT-Flag or Hsc70-3KA-Flag for 24 h, PD-L1 levels were detected by western blotting.
(H) MCF-7 cells were transfected with Hsc70-WT-Flag or Hsc70-3KA-Flag plasmids for 24 h. Fluorescence of PD-L1 on the surface of cell membrane was analyzed by flow cytometry, n=6.
(I-K) MCF-7 cells were transfected with Flag, Hsc70-WT-Flag or Hsc70-3KA-Flag for 8 h, the co-localization between RAB7A and PD-L1 (I), Lamp1 and PD-L1 (J), RAB11 and PD-L1 (K) were analyzed by Pearson correlation coefficient with three replicates (n=3).
For C, D, F and H, MCF-7 cells were seeded in 48-well plate with 6 replicates per group and subjected to the corresponding treatment, repeated independently three times and similar results were obtained. Data shown in A, B, E and G were repeated independently three times with similar results.
Data represent Mean±SEM, for C, D, F, H and I-K data, two-sided with adjustment of Tukey's multiple comparisons, one-way ANOVA, P value is indicated in the graph.
The numbers under the blots represent the value (the ratio to Tubulin) of grayscale quantification. Source data are provided as a Source Data file.

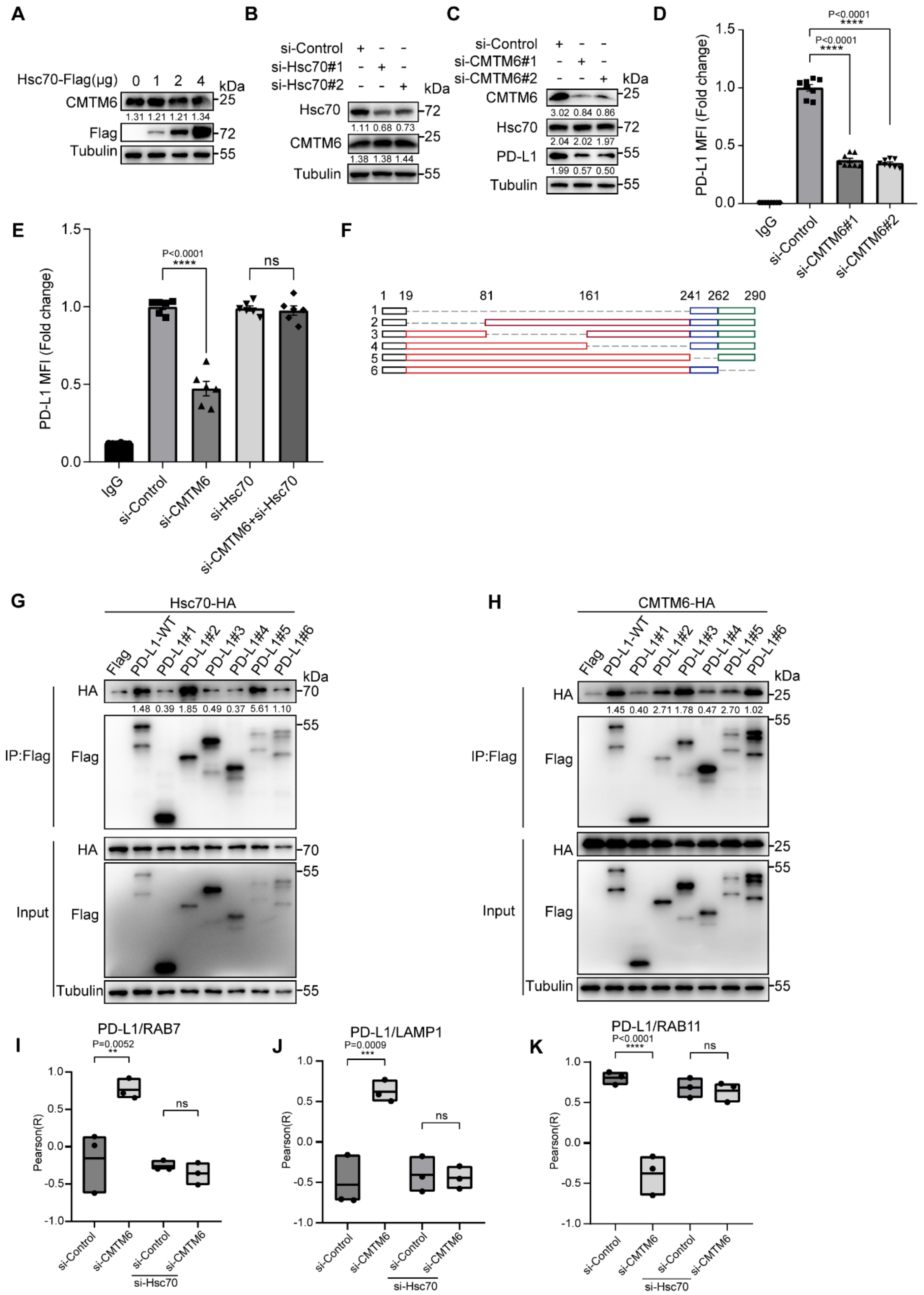

Supplementary Figure 3 PD-L1 degradation induced by CMTM6 depletion depends on Hsc70

(A) MCF-7 cells were transfected with 1 μg, 2 μg and 4 μg Hsc70-Flag for 24 h, CMTM6 levels were detected by western blotting.

(B) MCF-7 cells were transfected with siRNA of Hsc70 for 60 h, CMTM6 levels were detected by western blotting.

(C, D) MCF-7 cells were transfected with siRNA of CMTM6 for 60 h, PD-L1 and Hsc70 levels were detected by western blotting (C), fluorescence of PD-L1 on the surface of cell membrane was analyzed by flow cytometry, n=8 (D).
(E) MCF-7 cells were transfected with siRNA of Hsc70 and CMTM6 for 60 h, fluorescence of PD-L1 on the surface of cell membrane was analyzed by flow cytometry, n=6, ns: not significance.
(F) Schematic diagram of different truncation in PD-L1.
(G) HEK293T cells were transfected with indicated PD-L1-Flag and Hsc70-HA for 24 h, the interaction between PD-L1 and Hsc70 was analyzed by immunoprecipitation.
(H) HEK293T cells were transfected with indicated PD-L1-Flag and CMTM6-HA for 24 h, the interaction between PD-L1 and CMTM6 was detected by immunoprecipitation.
(I-K) MCF-7 cells were transfected with indicated siRNA for 36 h and the co-localization between RAB7A and PD-L1(I), LAMP1 and PD-L1 (J), RAB11 and PD-L1 (K) were analyzed by Pearson correlation coefficient with three replicates (n=3).
For D and E, MCF-7 cells were seeded in 48-well plate with 8 or 6 replicates per group and subjected to the corresponding treatment, repeated independently three times and similar results were obtained. Data shown in A-C and G-H were repeated independently three times with similar results.
Data represent Mean±SEM, for D, E and I-K data, two-sided with adjustment of Tukey's multiple comparisons, one-way ANOVA, P value is indicated in the graph.
The numbers under the blots represent the value (the ratio to Tubulin/Flag) of grayscale quantification. Source data are provided as a Source Data file.

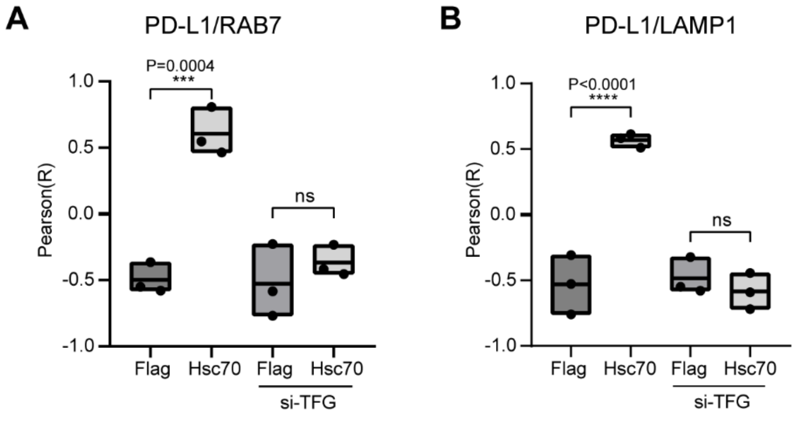

Supplementary Figure 4 TFG is involved in endosome-lysosome degradation of PD-L1

(A-B) MCF7 cells were transfected with siRNA of TFG for 48 h, then transfected with or without Hsc70 for another 8 h, the co-localization between RAB7A and PD-L1 (A), LAMP1 and PD-L1 (B) were analyzed by Pearson correlation coefficient with three replicates (n=3), ns: not significance. Data represent Mean±SEM, two-sided with adjustment of Tukey's multiple comparisons, one-way ANOVA, P value is indicated in the graph. Source data are provided as a Source Data file.

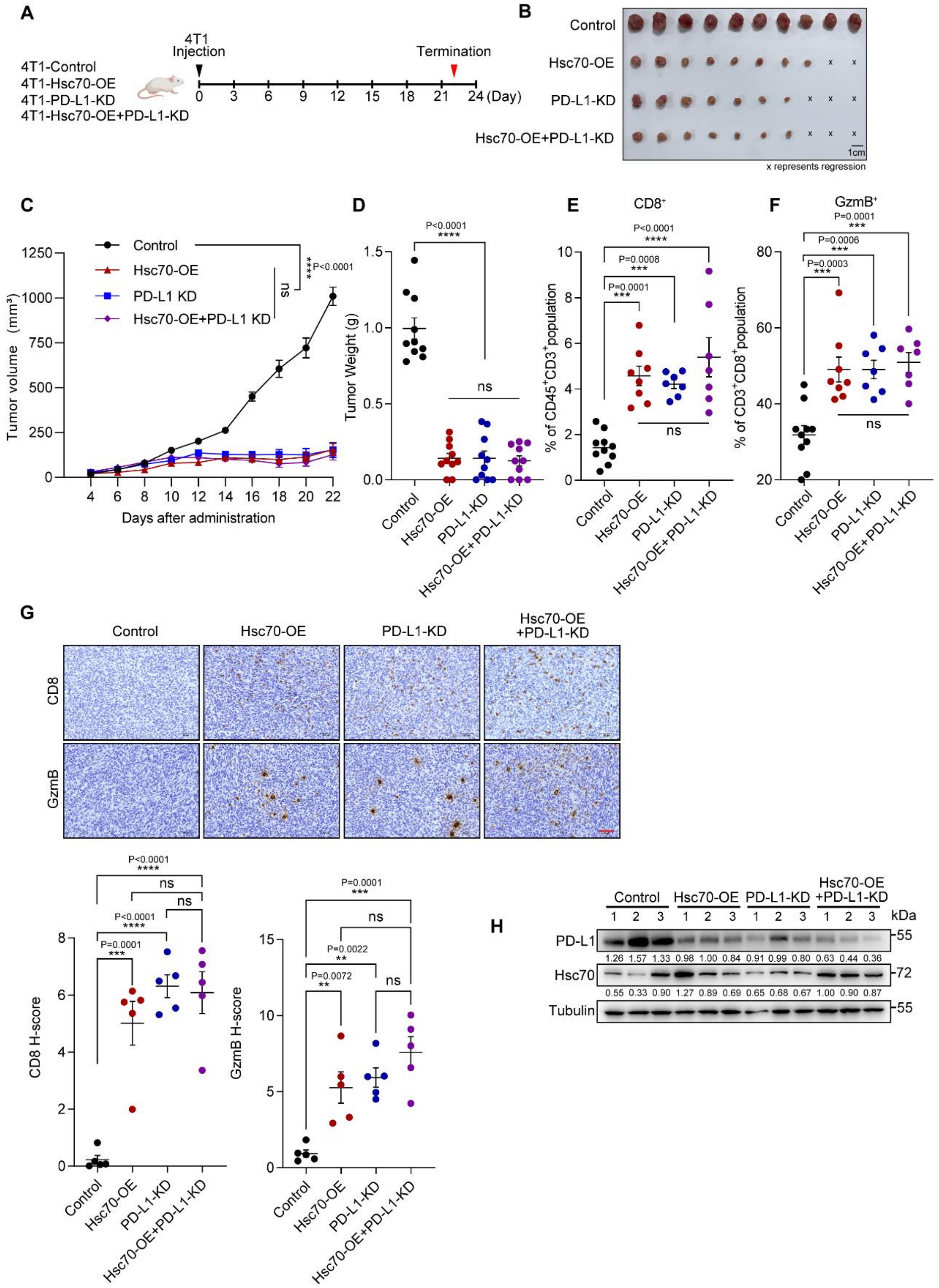

Supplementary Figure 5 The anti-tumor consequence of Hsc70 overexpression is specifically through PD-L1

(A) The schematic diagram of 4T1 breast cancer tumor model, created with BioRender.com, released under a Creative Commons Attribution-NonCommercial-NoDerivs 4.0 International license.

(B-D) Tumor growth of Control (n=10), PD-L1-KD (n=10), Hsc70-OE (n=10) and Hsc70-OE+PD-L1-KD (n=10) group which injected indicated cells in BALB/c mice and final tumor weights. (X represents tumor regression during the final dissection).

(E, F) Flow cytometry analysis of $CD8^+$ T cells (E) and $CD8^+GzmB^+$ T cells (F) in tumors. Control (n=10), PD-L1-KD (n=8), Hsc70-OE (n=7) and Hsc70-OE+PD-L1-KD (n=7).

(G) Immunohistochemistry analysis of CD8 and GzmB in tumors. Scale bar, 50 μm. n=5 (each group has 5 tumor tissues, every tissue has 3 random fields).

(H) Indicated protein levels were detected by Immunoblotting with the indicated harvested tumor cells. n=3 (randomly selected tumor tissues from 3 mice in each group for detection) and repeated independently two times with similar results.

Data represent Mean±SEM, for C two-way ANOVA, for D-G one-way ANOVA, two-sided with adjustment of Tukey's multiple comparisons, P value is indicated in the graph. ns: not significance.

The numbers under blots represent the value (the ratio to Tubulin) of grayscale quantification. Source data are provided as a Source Data file.

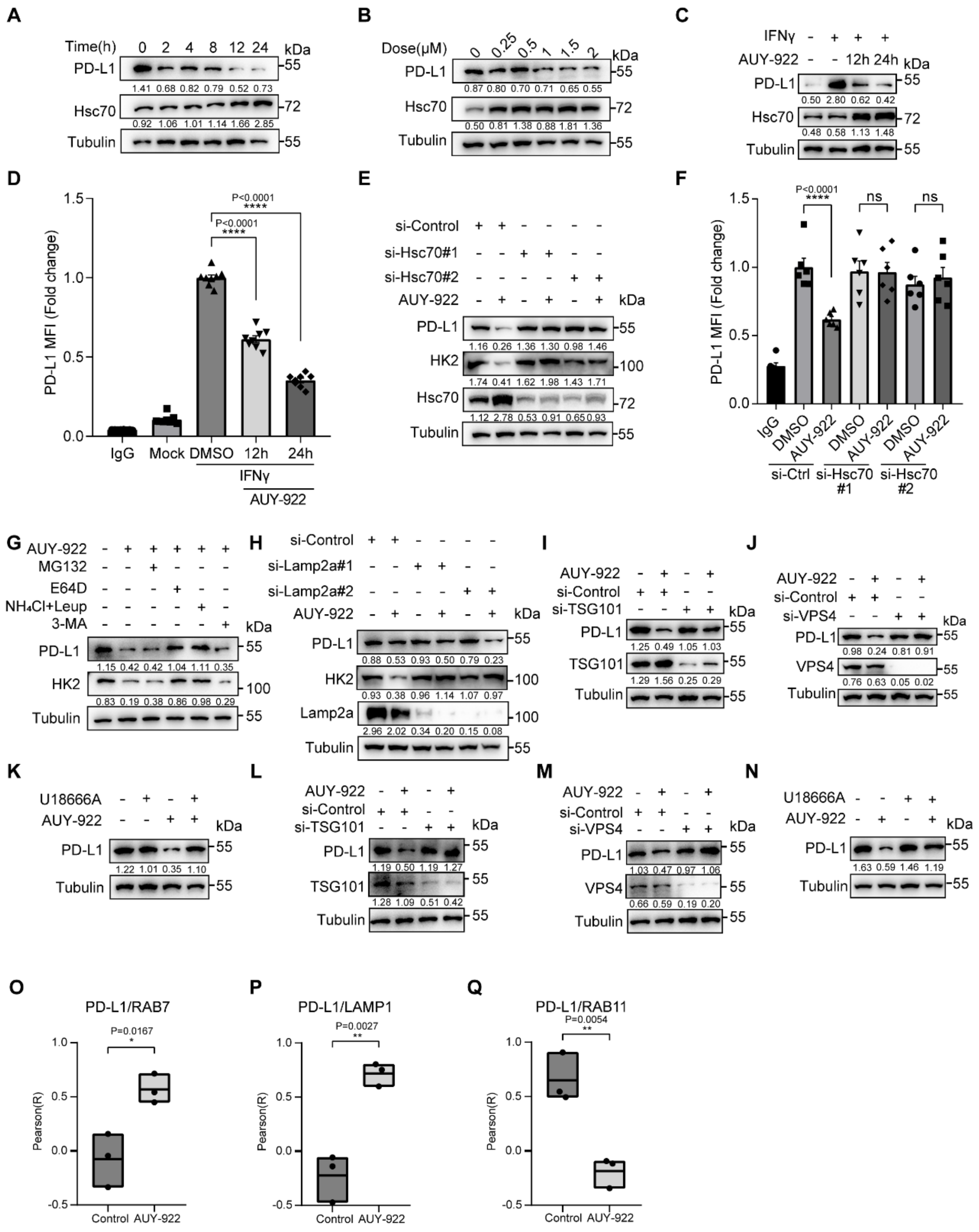

Supplementary Figure 6 AUY-922 induces PD-L1 degradation through eMI

(A) PANC1 cells were treated with 1 μM AUY-922 for 2, 4, 8, 12 and 24 h. Cell lysates were immunoblotted with indicated antibodies.
(B) PANC1 cells were treated with 0.25 μM, 0.5 μM, 1 μM, 1.5 μM or 2 μM AUY-922 for 24 h. Cell lysates were immunoblotted with indicated antibodies.
(C, D) U937 cells were pretreated with IFN-γ for 48 h, treated with 1 μM AUY-922 for another 12 h and 24 h. PD-L1 levels were detected by western blotting (C), fluorescence of PD-L1 on the surface of cell membrane was analyzed by flow

cytometry, n=8 (D).

(E, F) MCF-7 cells were transfected with siRNAs of Hsc70 for 48 h, treated with or without 1 μM AUY-922 for another 12 h. Cell lysates were immunoblotted with indicated antibodies (E), fluorescence of PD-L1 on the surface of cell membrane was analyzed by flow cytometry, n=6, ns: not significance (F).

(G) PANC1 cells were treated with 1 μM AUY-922 for 12 h, treated with or without MG132 (10 μM), NH_4Cl (20 mM), Leupeptin (100 nM), 3-MA (5 mM), E64D (10 μM) for another 12 h. Cell lysates were immunoblotted with indicated antibodies.

(H) PANC1 cells were transfected with siRNAs of Lamp2a for 48 h, treated with or without 1 μM AUY-922 for another 12 h. Cell lysates were immunoblotted with indicated antibodies.

(I) MCF-7 cells were transfected with siRNAs of TSG101 for 48 h, treated with or without 1 μM AUY-922 for another 12 h. Cell lysates were immunoblotted with indicated antibodies.

(J) MCF-7 cells were transfected with siRNAs of VPS4 for 48 h, treated with or without 1 μM AUY-922 for another 12 h. Cell lysates were immunoblotted with indicated antibodies.

(K) MCF-7 cells were treated with 1 μM AUY-922 with or without 5 μg/mL U18666A for 12 h. PD-L1 levels were detected by western blotting.

(L) PANC1 cells were transfected with siRNAs of TSG101 for 48 h, treated with or without 1 μM AUY-922 for another 12 h. Cell lysates were immunoblotted with indicated antibodies.

(M) PANC1 cells were transfected with siRNAs of VPS4 for 48 h, treated with or without 1 μM AUY-922 for another 12 h. Cell lysates were immunoblotted with indicated antibodies.

(N) PANC1 cells were treated with 1 μM AUY-922 with or without 5 μg/mL U18666A for 12 h. PD-L1 levels were detected by western blotting.

(O-Q) MCF-7 cells were treated with 1 μM AUY-922 for 4 h and the co-localization between RAB7A and PD-L1 (O), the co-localization between LAMP1 and PD-L1 (P), the co-localization between RAB11 and PD-L1 (Q) were analyzed by Pearson correlation coefficient with three replicates (n=3).

For D and F, U937 or MCF-7 cells were seeded in 48-well plate with 8 or 6 replicates per group and subjected to the corresponding treatment, repeated independently three times and similar results were obtained. Data shown in A-C, E and G-N were repeated independently three times with similar results.

Data represent Mean±SEM, for D, F one-way ANOVA, for O-Q t-test, two-sided with adjustment of Tukey’s multiple comparisons, P value is indicated in the graph.

The numbers under blots represent the value (the ratio to Tubulin) of grayscale quantification. Source data are provided as a Source Data file.

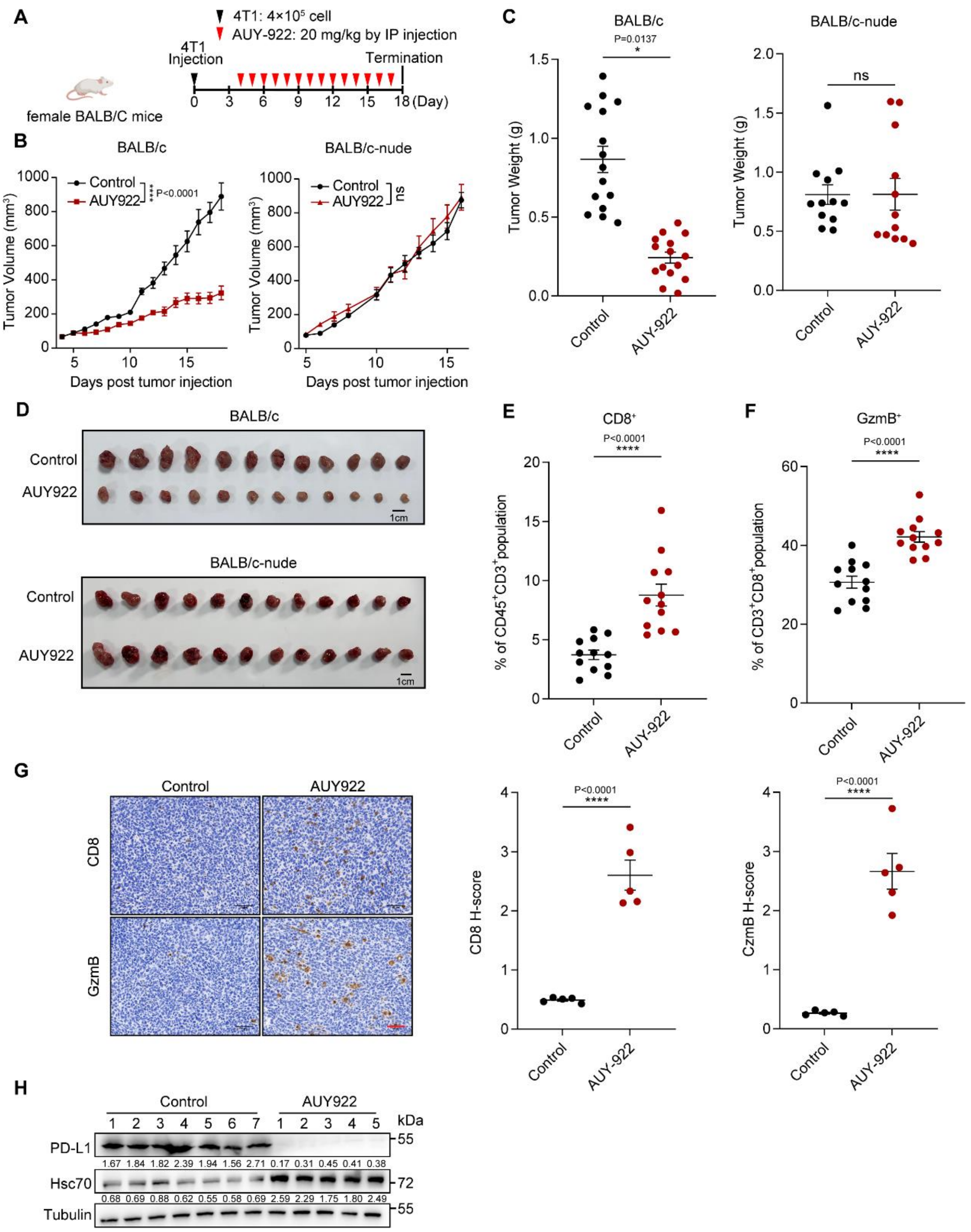

Supplementary Figure 7 AUY-922 inhibits tumor growth and promotes anti-tumor immunity

(A) BALB/c or BALB/c-nude mice were inoculated with 4×10^5 4T1 cells, group administration according to the time points shown in the schematic diagram, created with BioRender.com, released under a Creative Commons Attribution-NonCommercial-NoDerivs 4.0 International license.

(B-D) Tumor growth of Control (n=12), AUY-922 (20 mg/kg) (n=12) in BALB/c and BALB/c-nude mice and final tumor weights.

(E, F) Flow cytometry analysis of $CD8^+$ T cells (E) and $CD8^+GzmB^+$ T cells (F) in tumors of BALB/c mice. Control (n=12), AUY-922 (20 mg/kg) (n=12).

(G) Immunohistochemistry analysis of CD8 and GzmB in tumors. Scale bar, 50 μm. n=5 (each group has 5 tumor tissues, each tissue has 3 random fields).

(H) Indicated protein levels were detected by western blotting with indicated harvested tumor cells. n=7 or 5 (randomly selected tumor tissues from 7 or 5 mice in each group for detection) and repeated independently two times with similar results.

Data represent Mean±SEM, for B two-way ANOVA, for C, E-G t-test, P value is indicated in the graph, ns: not significance.

The numbers under blots represent the value (the ratio to Tubulin) of grayscale quantification. Source data are provided as a Source Data file.

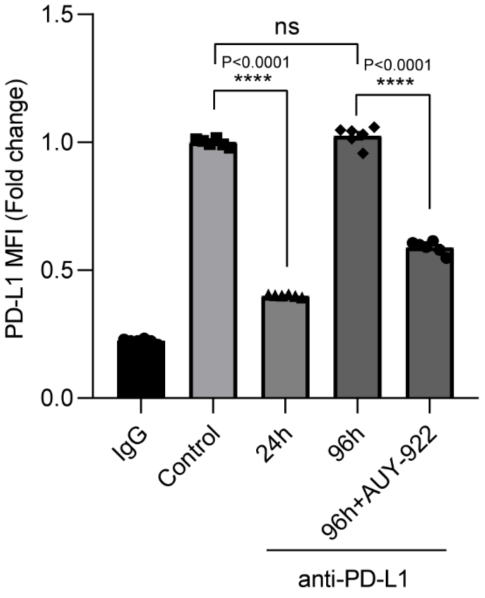

Supplementary Figure 8 AUY-922 promotes antitumor immunity and enhances the therapeutic effect of anti-PD-L1

4T1 cells were pretreated with IFN-γ (100 ng/mL) and treated with Anti-PD-L1 Ab (100 nM) for 24 h or 96 h, then treated with or without 1 μM AUY-922 for another 24 h, fluorescence of PD-L1 on the surface of cell membrane was analyzed by flow cytometry, n=6.

4T1 cells were seeded in 48-well plate with 6 replicates per group and subjected to the corresponding treatment, repeated independently three times and similar results were obtained. Data represent Mean±SEM, two-sided with adjustment of Tukey's multiple comparisons, P value is indicated in the graph, ns: not significance. Source data are provided as a Source Data file.

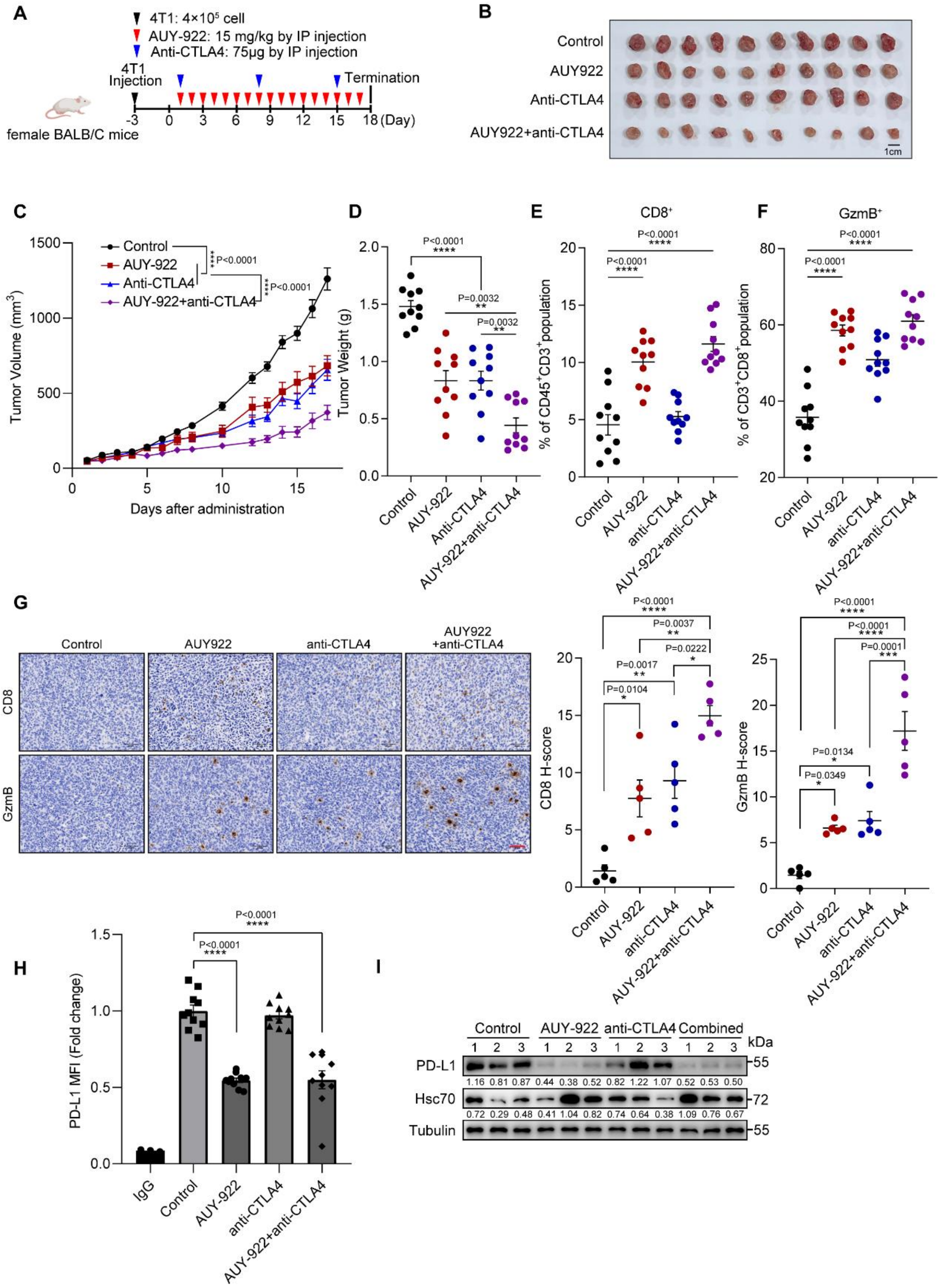

Supplementary Figure 9 AUY-922 promotes antitumor immunity and enhances the therapeutic effect of anti-CTLA4

(A) BALB/c mice were inoculated with 4×10^5 4T1 cells, group administration according to the time points shown in the schematic diagram, created with BioRender.com, released under a Creative Commons Attribution-NonCommercial-NoDerivs 4.0 International license.

(B-D) Tumor growth of Control (n=10), AUY-922 (15 mg/kg) (n=10), Anti-CTLA4 (75 μg) (n=10) and the combination of AUY-922 and anti-CTLA4 (AUY-922+anti-CTLA4) (n=10) in BALB/c mice and final tumor weights.
(E, F) Flow cytometry analysis of $CD8^+$ T cells (E) and $CD8^+GzmB^+$ T cells (F) in tumors. Control (n=10), AUY-922 (15 mg/kg) (n=10), Anti-CTLA4 (75 μg) (n=10) and AUY-922+anti-CTLA4 (n=10).
(G) Immunohistochemistry analysis of CD8 and GzmB in tumors. Scale bar, 50 μm. n=5 (each group has 5 tumor tissues, every tissue has 3 random fields).
(H) Flow cytometry analysis of PD-L1 levels on the surface of cell membrane in tumor cells. Control (n=10), AUY-922 (n=10), Anti-CTLA4 (n=10) and AUY-922+anti-CTLA4 (n=10).
(I) Indicated protein levels were detected by Immunoblotting with the indicated harvested tumor cells. n=3 (randomly selected tumor tissues from 3 mice in each group for detection) and repeated independently two times with similar results.
Data represent Mean±SEM, for C two-way ANOVA, for D-H one-way ANOVA, two-sided with adjustment of Tukey's multiple comparisons, P value is indicated in the graph.
The numbers under blots represent the value (the ratio to Tubulin) of grayscale quantification. Source data are provided as a Source Data file.

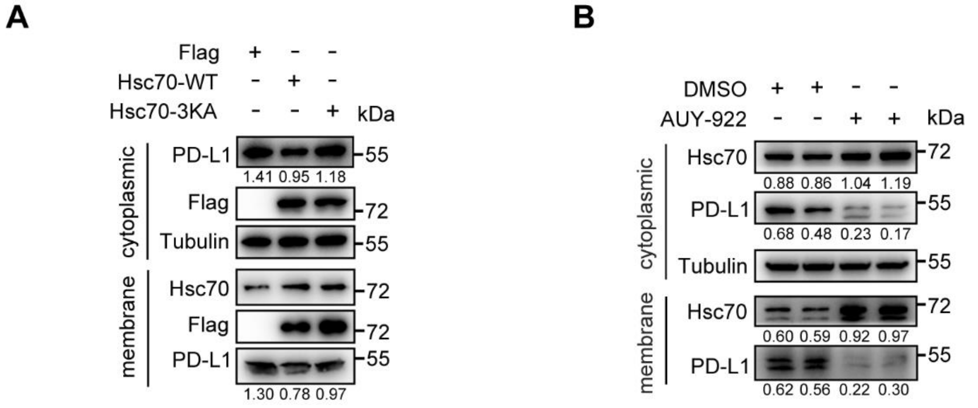

Supplementary Figure 10 Overexpression of Hsc70 or AUY-922 treatment enhanced the protein levels of Hsc70 on membrane and reduced the protein levels of PD-L1 on membrane and in the cytoplasmic

(A) MCF-7 cells were pretreated with IFN-γ for 48 h, then transfected with Flag, Hsc70-WT-Flag or Hsc70-3KA-Flag (CMV promoter) for 24 h, collect cells to extract cytoplasmic and membrane proteins, detected the protein levels of Tubulin, PD-L1, Flag by western blotting.
(B) MCF-7 cells were pretreated with IFN-γ for 48 h, then treated with or without AUY-922 for 12 h, collect cells to extract cytoplasmic and membrane proteins, detected the protein levels of Tubulin, PD-L1, Flag by western blotting.
Data shown in A and B were repeated independently three times with similar results.
The numbers under blots represent the value (the ratio to Tubulin) of grayscale

quantification. Source data are provided as a Source Data file.

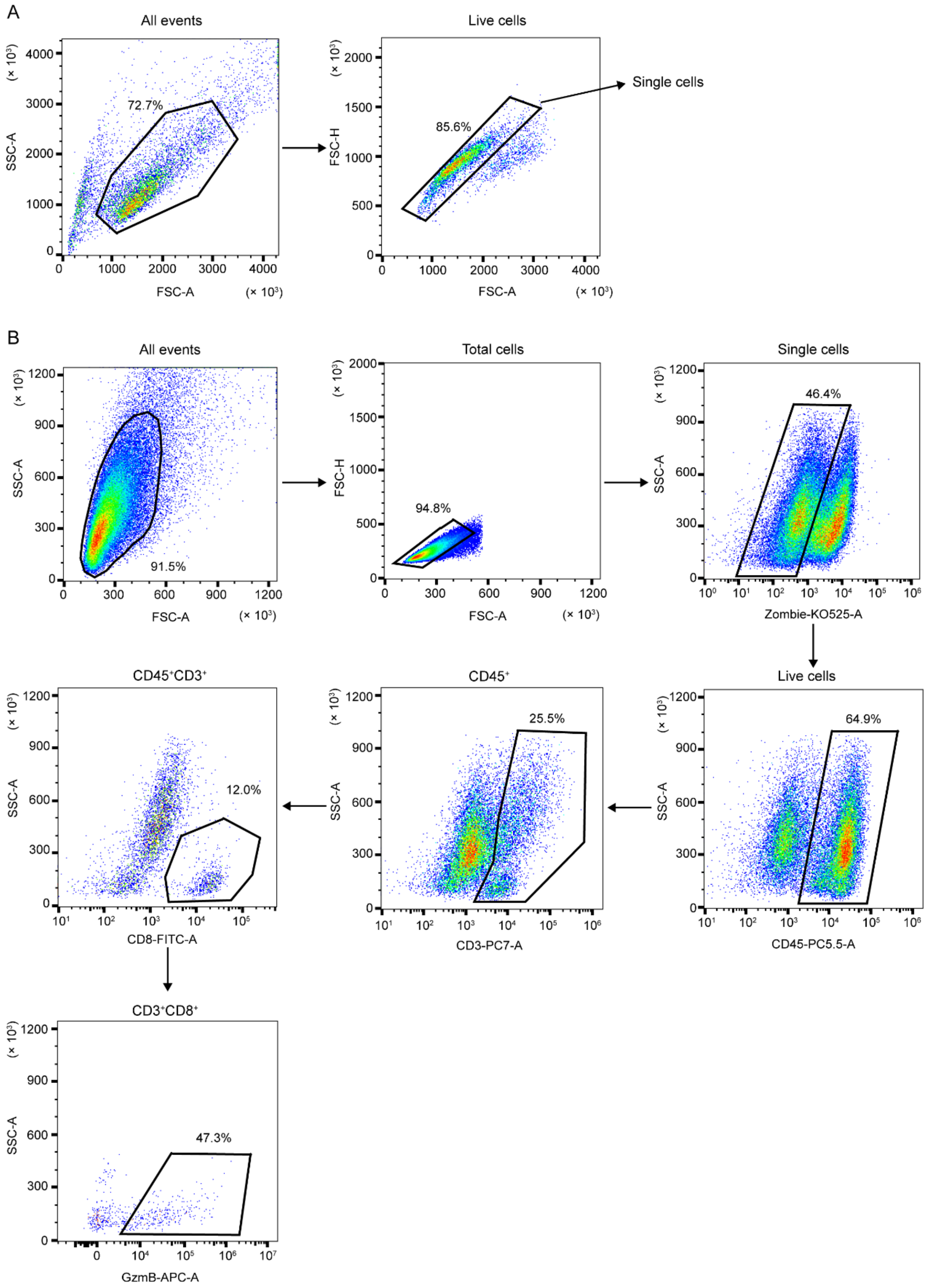

Supplementary Figure 11 Gating strategy of flow cytometry

(A) The schematic diagram of cell flow cytometry gating strategy. Data from Figure 3E si-

Control. Figure 1 C, F, H, J, Figure 2 B, D, F, H, Figure 3 B, E, G, I, Figure 4 H, Figure 6 C, E, G, H, I, Figure 7 H, Supplementary Figure 1 A, E, J, K, N, Supplementary Figure 2 C, F, H, Supplementary Figure 3 D, E, Supplementary Figure 6 D, F, Supplementary Figure 8, Supplementary Figure 9 H follow this strategy.

(B) The schematic diagram of tumor flow cytometry gating strategy. Data is from Figure 7E, F; tumor treated by anti-PD-L1. Figure 5 E, F, Figure 7 E, F, Supplementary Figure 5 E, F, Supplementary Figure 7 E, F, Supplementary Figure 9 E, F. follow this strategy. In detailed Zombie-Violet antibody was used to gate the living cells, PerCP-Cy5.5 conjugated-CD45 antibody was used to gate the immune cells, PE-Cy7 conjugated-CD3 antibody was used to gate the T cells, FITC conjugated-CD8 antibody was used to circle the cytotoxic T cells, and APC conjugated- Granzyme B antibody was used to gate the activated cytotoxic T cells. Source data are provided as a Source Data file.

Supplementary Table 1. The siRNA sequence use in this study

Genes	SiRNA sequence
si-Control	5’-UUCUCCGAACGUGUCACGUTT-3’
si-Hsc70	5’-GGCCAGUAUUGAGAUCGAUTT-3’
si-Lamp2a#1	5’-GGAGCAUUUCAGAUAAAUAUU-3’
si-Lamp2a#2	5’-GCAUGUUGGUGGAAAGAUAAU-3’
si-CMTM6	5’-CCCAAGACAGUGAAAGUAAUU-3’
si-TSG101	5’-CUAGUUCAAUGACUAUUAAUU-3’
si-VPS4	5’-CCGAGAAGCUGAAGGAUUAUU-3’
si-TFG#1	5’-GAUAGAACUUCGAAAUAAATT-3’
si-TFG#2	5’- GGUAUUCAGUAUUCAGCAATT-3’
sh-PD-L1	GCAGGCGTTTACTGCTGCATATTCAAGAGATATGCAGCAGTAAA CGCCTGCTTTTTT

Supplementary Table 2. Primer sequences for Real-time PCR

Genes	Primer-F (5’-3’)	Primer-R (5’-3’)
Tubulin	TGGTAGAGAATACTGATGAGAC	GCTGAGACAAGGTGGTTC
PD-L1	GCACACTGAGAATCAACAC	GCTACACCAAGGCATAATAAG
Hsc70	TGCTGTGGACAAGAGTACGG	GTCTTCCTTGCTCAAACGGC
