# Supplementary material for: Hsc70 promotes anti-tumor immunity by targeting PD-L1 for lysosomal degradation
Source: Nat Commun. 2024 May 18;15:4237. doi: 10.1038/s41467-024-48597-3 (PMC11102475; doi:10.1038/s41467-024-48597-3)
Supplement: Supplementary file 3 — Reporting Summary [file 41467_2024_48597_MOESM3_ESM.pdf]

nature portfolio

Corresponding author(s): Hongguang Xia
Last updated by author(s): Apr 24, 2024

Reporting Summary

Nature Portfolio wishes to improve the reproducibility of the work that we publish. This form provides structure for consistency and transparency in reporting. For further information on Nature Portfolio policies, see our Editorial Policies and the Editorial Policy Checklist.

Statistics

For all statistical analyses, confirm that the following items are present in the figure legend, table legend, main text, or Methods section.

n/a	Confirmed	
[ ]	[x]	The exact sample size (*n*) for each experimental group/condition, given as a discrete number and unit of measurement
[ ]	[x]	A statement on whether measurements were taken from distinct samples or whether the same sample was measured repeatedly
[ ]	[x]	The statistical test(s) used AND whether they are one- or two-sided<br>*Only common tests should be described solely by name; describe more complex techniques in the Methods section.*
[x]	[ ]	A description of all covariates tested
[x]	[ ]	A description of any assumptions or corrections, such as tests of normality and adjustment for multiple comparisons
[ ]	[x]	A full description of the statistical parameters including central tendency (e.g. means) or other basic estimates (e.g. regression coefficient) AND variation (e.g. standard deviation) or associated estimates of uncertainty (e.g. confidence intervals)
[ ]	[x]	For null hypothesis testing, the test statistic (e.g. *F*, *t*, *r*) with confidence intervals, effect sizes, degrees of freedom and *P* value noted<br>*Give P values as exact values whenever suitable.*
[x]	[ ]	For Bayesian analysis, information on the choice of priors and Markov chain Monte Carlo settings
[x]	[ ]	For hierarchical and complex designs, identification of the appropriate level for tests and full reporting of outcomes
[ ]	[x]	Estimates of effect sizes (e.g. Cohen's *d*, Pearson's *r*), indicating how they were calculated

Our web collection on statistics for biologists contains articles on many of the points above.

Software and code

Policy information about availability of computer code

Data collection	Flow cytometry data were collected using CytExpert v2.4 (Beckman Coulter).<br>Imaging data were collected using Olympus FV3000 Laser Scanning Confocal Microscope.
Data analysis	GraphPad Prism 8.0 was used for statistical analyses.<br>FV31S-SW and ImageJ 1.44p were used for immunofluorescence images processing.<br>K-Viewer was used for immunohistochemistry images processing.<br>CytExpert v2.4 and FlowJo v10.8.1 was used for the analysis and quantification of flow cytometry.<br>MaxQuant v1.5 was used for protein identification and quantification in mass spectrometry data.<br>Kingdraw v2.1.0 was used for chemical structure drawing.

For manuscripts utilizing custom algorithms or software that are central to the research but not yet described in published literature, software must be made available to editors and reviewers. We strongly encourage code deposition in a community repository (e.g. GitHub). See the Nature Portfolio guidelines for submitting code & software for further information.

Data

Policy information about availability of data

All manuscripts must include a data availability statement. This statement should provide the following information, where applicable:

- Accession codes, unique identifiers, or web links for publicly available datasets
- A description of any restrictions on data availability
- For clinical datasets or third party data, please ensure that the statement adheres to our policy

Data Availability
The mass spectrometry proteomics data generated in this study have been deposited in the ProteomeXchange Consortium (https://proteomecentral.proteomexchange.org) via the iProX partner repository with the dataset identifier PXD051281 (https://www.iprox.cn//page/project.html?id=IPX0008562000), PXD051241 (https://www.iprox.cn//page/project.html?id=IPX0008528000). The remaining data are available within the Article, Supplementary Information or Source Data file. Source data are provided with this paper.

Research involving human participants, their data, or biological material

Policy information about studies with human participants or human data. See also policy information about sex, gender (identity/presentation), and sexual orientation and race, ethnicity and racism.

Reporting on sex and gender	No human participants or human data were included.
Reporting on race, ethnicity, or other socially relevant groupings	No human participants or human data were included.
Population characteristics	No human participants or human data were included.
Recruitment	No human participants or human data were included.
Ethics oversight	No human participants or human data were included.

Note that full information on the approval of the study protocol must also be provided in the manuscript.

Field-specific reporting

Please select the one below that is the best fit for your research. If you are not sure, read the appropriate sections before making your selection.

☒ Life sciences ☐ Behavioural & social sciences ☐ Ecological, evolutionary & environmental sciences

For a reference copy of the document with all sections, see nature.com/documents/nr-reporting-summary-flat.pdf

Life sciences study design

All studies must disclose on these points even when the disclosure is negative.

Sample size	Sample size estimates has been performed on previous experience to obtain statistical significance and reproducibility. For in vitro experiments such as Western blot, qPCR, IHC, IF and flow cytometry, at least three samples were used per group for minimal statistics requirements. For in vivo studies, we chose a sample size of no less than 10 animals per group, which is sufficient to obtain statistical differences between groups.
Data exclusions	There are no data exclusions.
Replication	All experiments underlying the main conclusions of this study, including Western blot, qPCR, IHC, IF, flow cytometry and in vivo experiments have been successfully replicated at least two to three times, yielding consistent results. Furthermore, these experimental findings have been corroborated by multiple models.
Randomization	Samples and organisms were randomly allocated to experimental groups. No specific randomization protocol has been used. Mice were age- and sex matched.
Blinding	No specific blinding was applied since all experiments were assigned into groups including relevalant controls and analysis was done objectively and without bias.

Reporting for specific materials, systems and methods

We require information from authors about some types of materials, experimental systems and methods used in many studies. Here, indicate whether each material, system or method listed is relevant to your study. If you are not sure if a list item applies to your research, read the appropriate section before selecting a response.

Materials & experimental systems

n/a	Involved in the study
	[x] Antibodies
	[x] Eukaryotic cell lines
[x]	Palaeontology and archaeology
	[x] Animals and other organisms
[x]	Clinical data
[x]	Dual use research of concern
[x]	Plants

Methods

n/a	Involved in the study
[x]	ChIP-seq
	[x] Flow cytometry
[x]	MRI-based neuroimaging

Antibodies

Antibodies used

The antibodies were provided as follows: HK2 (#22029-1-AP, 1:1000, Proteintech), PD-L1 (#66248-1-lg, 1:1000, Proteintech), Hsc70 (#10654-1-AP, 1:2000, Proteintech), β-tubulin (#M1305-2, 1:5000, HUABIO), Flag-Tag (#M1403-2, 1:2000, HUABIO), HA-Tag (#0906-1, 1:2000, HUABIO), Lamp2a (#ab18528, 1:1000, Abcam), TSG101 (#ab83, 1:1000, abcam), VPS4 (#ab229806, 1:1000, abcam), CMTM6 (#HPA026980, 1:1000, Sigma), TFG (#ab156866, 1:1000, Abcam).
The following antibodies were used in immunofluorescence: LAMP1 (#D4O1S, 1:100, Cell Signaling Technology), RAB7A (#E9O7E, 1:100, Cell Signaling Technology), PD-L1 (#D8T4X, 1:100, Cell Signaling Technology), RAB11 (#610656, 1:50, BD Science).
The antibodies for immunohistochemistry (IHC) were used: CD8α (#98941, 1:200, Cell Signaling Technology), Granzyme B (#44153, 1:100, Cell Signaling Technology).
The following antibodies for flow cytometry analysis were displayed: PE anti-human CD274 (29E.2A3) (#329706, 1:200, Biolegend), PE Mouse IgG2b, κ Isotype Ctrl (MPC-11) (#400312, 1:200, Biolegend), PE anti-mouse CD274 (MIH6) (#153612, 1:200, Biolegend), PE Rat IgG2a, λ Isotype Ctrl (G013C12) (#402304, Biolegend), Zombie Violet™ Fixable Viability Kit (#423114; 1:200; Biolegend), PerCP/Cyanine5.5 anti-mouse CD45 (#103132; 1:200; Biolegend), PE/Cyanine7 anti-mouse CD3 (#100320; 1:200; Biolegend), FITC anti-mouse CD8 (#100706; 1:200; Biolegend), APC anti-human/mouse Granzyme B (#372204; 1: 200; Biolegend), APC/Cyanine7 anti-mouse CD8a (#100714; 1:200; Biolegend), PE anti-rat IgG2b (#408214, 1:100, Biolegend).
The secondary antibodies for western blot were used: goat anti-mouse (#31430,1:20000, Thermo Fisher Scientific), goat anti-rabbit (#31460, 1:20000, Thermo Fisher Scientific).
The fluorescent secondary antibodies for immunofluorescence were used: goat anti-rabbit Alexa Fluor 546 (#A-11035, 1:500, Thermo Fisher Scientific), goat anti-mouse Alexa Fluor 488 (#A-11029, 1:500, Thermo Fisher Scientific), goat anti-mouse DyLight 649 (#A23610, 1:500, Abbkine).
The in vivo antibodies for mouse models were used: control antibody InVivoMab rat IgG2b isotype (#BE0090; 100μg; Bioxcell), anti-PD-L1 (#BE0101; 100μg; Bioxcell), anti-CTLA4 Ab (#BE0131; 75 μg; Bioxcell).

Validation

For all the antibodies, we carried out western blot according to the method on the company's website, and detected whether the band size met the expectation with molecular weight marker, and added appropriate positive control and negative control. For human Hsc70, TSG101, VPS4, CMTM6 and TFG, we added siRNA knockdown verification. Antibodies purchased from Cell Signaling Technology were validated as per their website stating "Antibody signal is measured in model systems with known presence/absence of target signal. Includes wild-type vs. genetic knockout, targeted induction or silencing." Besides, each antibody's manual contains authentic data results from the companies (HUABIO, Abcam, Proteintech and Sigma) validating specificity, and our data also verifies the corresponding antibody's specificity.

Eukaryotic cell lines

Policy information about cell lines and Sex and Gender in Research

Cell line source(s)

HEK293T (ATCC CRL-3216), MCF-7 (ATCC HTB-22), and PANC1 (ATCC CRL-1469) cells were grown in DMEM medium (Hyclone, with L-glutamine, with 4.5 g/L glucose, without pyruvate); U937 (CTCC-001-0027, Meisen) and 4T1 (ATCC CRL-2539) cells were obtained from ATCC and Meisen. PD-L1-KD stable cell, PD-L1-KD+Hsc70-OE stable cell lines, Hsc70-WT-OE stable cell lines and Hsc70-3KA-OE stable cell lines were generated by our laboratory through lentivirus transduction.

Authentication

All cell lines were authenticated by STR profiling

Mycoplasma contamination

All used cell lines were tested negative for contamination.

Commonly misidentified lines (See ICLAC register)

None of commonly misidentified cell lines has been used.

Animals and other research organisms

Policy information about studies involving animals; ARRIVE guidelines recommended for reporting animal research, and Sex and Gender in Research

Laboratory animals

Female BALB/c mice and nude mice (aged 8-10 weeks) were purchased from Shanghai SLAC Laboratory Animal Co., Ltd. (Shanghai, China). All the animal experiments were strictly conducted in accordance with the protocols approved by the Ethics Committee for Animal Studies at Zhejiang University, China. All mice were cultured in suitable temperature and humidity environment (25°C, suitable humidity (typically 50%) ,12 hour dark/light cycle), and fed with sufficient water and food .

Wild animals	We don't use wild animals.
Reporting on sex	We used female BALB/c and nude mice. Since breast cancer mainly occurs in women and is the leading cause of cancer deaths among women, female mice are selected for modeling. We used 4T1 cells in female mice to mimic human triple-negative breast cancer (TNBC). 4T1 cells can be transplanted into the fat pad of the mouse mammary gland, in contrast to male mice, these tumor cells are highly tumorigenic, invasive, and spontaneously metastatic. Experiments with 4T1 cells in female mice could provide a valuable model system for preclinical TNBC studies.
Field-collected samples	No field collected samples were used in the study.
Ethics oversight	All mice were housed in a specific pathogen-free (SPF) facility and were euthanized with carbon dioxide. All the animal experiments were strictly conducted in accordance with the protocols approved by the Tab of Animal Experimental Ethical Inspection of the First Affiliated Hospital, College of Medicine, Zhejiang University. The tumor size allowed in the experiment did not exceed 2000mm3, we adhere to this limit to ensure that the animals did not suffer undue harm and that the experimental results are valid and reproducible.

Note that full information on the approval of the study protocol must also be provided in the manuscript.

Flow Cytometry

Plots

Confirm that:

☒ The axis labels state the marker and fluorochrome used (e.g. CD4-FITC).

☒ The axis scales are clearly visible. Include numbers along axes only for bottom left plot of group (a 'group' is an analysis of identical markers).

☒ All plots are contour plots with outliers or pseudocolor plots.

☒ A numerical value for number of cells or percentage (with statistics) is provided.

Methodology

Sample preparation	Tumors were collected and processed into single-cell suspensions through digestion in collagenase type I (#2350118, Gibco) and DNase I (#143582, Roche) at 37°C for 45 min. After filtering with a 45 μm filter (BD Bioscience), the isolated cells were stained with the specific surface marker antibodies, Zombie Violet™ Fixable Viability Kit (#423114; Biolegend), anti-CD45-PerCP-Cy5.5 (#103132; Biolegend), anti-CD3-PE-Cy7(#100320; Biolegend) and anti-CD8-FITC (#100706; Biolegend) in PBS for 30 min at 4°C. Intracellular staining of GzmB was performed as follows: cells were washed and then fixed and permeabilized with a Fix/Perm kit (#421403; Biolegend), and finally stained with anti-APC-GzmB (#372204; Biolegend). For proper compensation of flow cytometry channels, single-stain samples were utilized.<br>For flow cytometric analysis of membrane PD-L1, 4T1, U937 or MCF-7 cells were collected by centrifugation at 1000 × g for 5 min, incubated with PBS (0.5% BSA) for 10 min at room temperature. The cells were probed with PE anti-mouse CD274 (MIH6) (#153612, 1:200, Biolegend) or PE anti-human CD274 (29E.2A3) (#329706, Biolegend) and a matched isotype at 4°C for 30 min in the dark. After washing three times with PBS, the cells were analyzed using flow cytometry (Beckman Coulter Cytoflex), and data were analyzed using software CytExpert2.4 and FlowJo v10.8.1.
Instrument	CytoFlex analyzer (Beckman Coulter)
Software	CytExpert v2.4, FlowJo v10.8.1 and GraphPad Prism 8.0
Cell population abundance	moderate
Gating strategy	In our experiment, Zombie-Violet antibody was used to gate the living cells, PerCP-Cy5.5 conjugated-CD45 antibody was used to gate the immune cells, PE-Cy7 conjugated-CD3 antibody was used to gate the T cells, FITC conjugated-CD8 antibody was used to circle the cytotoxic T cells, and APC conjugated-GzmB antibody was used to gate the activated cytotoxic T cells.

☒ Tick this box to confirm that a figure exemplifying the gating strategy is provided in the Supplementary Information.
